# Positions of the horizontal and vertical centre of rotation in eyes with different refractive errors

**DOI:** 10.1111/opo.12940

**Published:** 2022-01-20

**Authors:** Arne Ohlendorf, Frank Schaeffel, Siegfried Wahl

**Affiliations:** ^1^ Carl Zeiss Vision International GmbH Technology & Innovation Aalen Germany; ^2^ Section of Neurobiology of the Eye Ophthalmic Research Institute University of Tuebingen Tuebingen Germany; ^3^ ZEISS Vision Science Lab Ophthalmic Research Institute University of Tuebingen Tuebingen Germany

**Keywords:** axial length, centre of rotation, line of sight, refractive error, spectacle lens

## Abstract

**Purpose:**

To determine how the position of the centre of rotation of the eyeball is related to axial length and refractive error when horizontal and vertical eye movements are performed.

**Methods:**

A custom‐built eye tracker was used that determined the centre of rotation of the eye (COR) from lateral displacements of the pupil centre. Horizontal and vertical eye movements were studied in the right eyes, and each measurement performed five times in 59 subjects (32 females) with an average age of 36.6 ± 9.1 years. Spherical equivalent refractive errors ranged from −9.7 to +6.8 D with an average error of −1.5 ± 2.9 D. Axial lengths were measured with the ZEISS IOL Master 500.

**Results:**

The mean horizontal centre of rotation (COR) of the right eye for a saccade from 0° to ±11.9° was 15.3 ± 1.5 mm behind the corneal apex, while the average vertical COR for the same angle of eccentricity was 12.5 ± 1.4 mm, indicating that the horizontal COR was 2.8 ± 1.7 mm behind the vertical COR. In right eyes, horizontal COR was significantly correlated with axial length (*r* = 0.28, *p* = 0.02) but not with the spherical equivalent refractive error (*r* = 0.39, *p* = 0.90). Similarly, vertical COR was significantly correlated with axial length (*r* = 0.25, *p* = 0.03) but not with the spherical equivalent refractive error (*r* = 0.17, *p* = 0.90).

**Conclusions:**

While it might be expected that the COR is dependent on axial length, the correlation was not strong. Interestingly, the location of the COR was substantially different for horizontal and vertical eye movements which may relate to the flatter curvature of the eyeball in the vertical meridian, compared to the horizontal, as described in previous studies.

**Key points**

- The centre of rotation for horizontal eye movements is about 15.3 mm behind the corneal apex while the centre of rotation for vertical eye movements is about 2.8 mm more anterior.
- Knowledge of the position of the centre of rotation is important for the optical design of spectacle lenses.
- In our subject sample (*n* = 59) with refractive errors ranging from −9.7 to 6.8 D, the position of the centre of rotation was not dependent on axial length.

## INTRODUCTION

### The centre of rotation of the eye

Our eyes move continuously to keep the images of interesting visual targets on the fovea. Eye movements can be divided into gaze‐stabilising and gaze‐shifting movements.^1^ All movements or rotations of the eyeball are performed around a centre of rotation (COR) that is located inside the globe, behind the posterior pole of the lens and close to the posterior nodal distance.^2^ Since the globe is not perfectly spherical, the exact location of the COR is somewhat controversial. For simplicity, a stationary centre of rotation is assumed that may shift nasally relative to the intersection lines of the fixation axes.^3^

Empirical studies suggested that the COR, on average, is located 13–15 mm behind the corneal vertex of the eye.^2^, ^3^, ^4^ Because the emmetropic eye is assumed to be close to spherical, the COR during eye movements should coincide with the centre of curvature of the posterior globe.^5^ However, when eye shape and axial length change due to the presence of axial refractive errors, this may change. For example, Grolman^2^ found that every dioptre of ametropia changes the position of the COR by 0.14 and 0.18 mm in the horizontal and vertical planes, respectively, as measured from the corneal vertex. Fry and Hill^6^ observed differences between the horizontal and vertical centres of rotation, and concluded that the COR for vertical movements of the eye is located in front of the COR for horizontal eye movements.

### Role of the COR in spectacle lens design

Unlike contact lenses or intraocular lenses which move with the eye, a spectacle lens is stationary in the visual field. Its position is described by the back vertex distance (the distance between the spectacle lens and the corneal apex) and its centration, relative to the line of fixation when the subjects looks straight forward. The COR determines where the line of fixation penetrates the spectacle lens when the peripheral visual field of view is explored, and it also has implications for peripheral lens design. The design should take the position of the COR into account since it determines the contribution of each region of the lens to the formation of the retinal image. Perches^7^ calculated visual acuity maps as a function of base curve and the position of the centre of rotation of the eye, using numerical ray tracing. Especially in high powered positive spherical lenses, the authors observed degraded image quality for oblique gaze, no matter what centre of rotation or base curve was evaluated.

Since the position of the COR in the eye is important, the current study investigated how refractive errors and the axial length of the eye determine the position of the COR during horizontal and vertical eye movements using a custom‐built eye tracker.

## METHODS

### Subjects

Fifty nine subjects (32 females) with an average age of 36.6 ± 9.1 years participated in the experiments. Thirty one subjects were classified as myopes (spherical equivalent refractive error (SE) ≤ −0.50 D), 18 as emmetropes (SE between −0.5 and +0.5 D) and 10 as hyperopes (SE > +0.50 D). Objective refraction was measured with a wavefront aberrometer (i. Profiler plus, Carl Zeiss Vision, zeiss.com) and axial length was measured with the ZEISS IOL Master 500 (Carl Zeiss Meditec, zeiss.com).

### Custom‐built eye tracker to determine the COR

**FIGURE 1:**
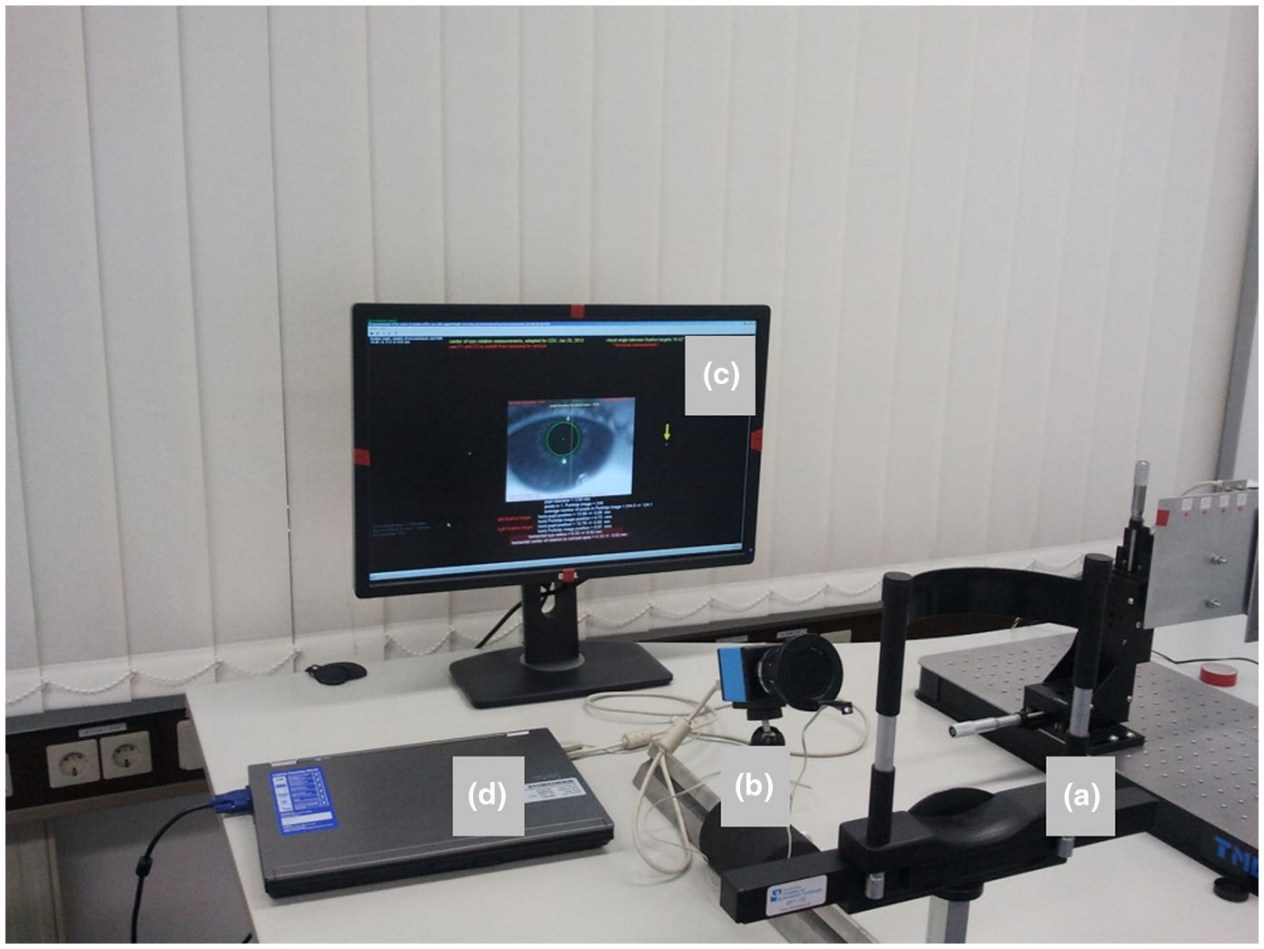
Setup to measure the horizontal and vertical centre of rotation. (a) Chin and headrest, (b) Camera and LED, (c) Monitor to display targets and (d) Laptop computer to run software

The COR of the eye was determined by measuring how much the pupil centre was displaced laterally when the subject made a saccade towards a peripheral fixation target that appeared on a computer screen at a distance of 80 cm. Figure 1 shows the setup of the measurement: a chin rest with a tightly stabilised head position was required. A monochrome USB camera, equipped with a 50 mm f/1.4 lens with an infrared light transmitting filter recorded an image of the black pupil at 62 Hz from a distance of 240 mm. A high‐power infrared LED, attached to the lens at an eccentricity of 40 mm served as the light source. The software detected the pupil by an adjustable thresholding procedure (typically, pixels were counted that were darker than 60% of the average pixel value in the video frame). The pupil centre was located simply by the centre of mass of the dark pixels.

To identify episodes where the subject fixated the visual targets, the software continuously determined the standard deviation of pupil centre positions from the latest 25 measurements (equivalent to about 0.5 s). Fixation was assumed when the running standard deviation dropped below 0.5°. In this case, the software stored the pupil positions, emitted a beep and presented a new fixation target. From the movement of the pupil centre and knowledge of the visual angle subtended by two fixation points, the COR of the eye from the plane of the entrance pupil could be automatically determined. One would prefer to determine this distance as measured from the corneal apex rather than from the plane of the entrance pupil. Fortunately, the plane of focus of the first Purkinje image, generated by the infrared light‐emitting diode, was calculated to be behind the corneal surface at a depth of half the corneal radius of curvature.^8^ As described previously based on published literature, a value for the corneal radius of curvature was assumed (R = 7.6 mm), providing a plane of focus 3.8 mm behind the corneal apex. The literature states that the plane of the entrance pupil is 3.6 mm behind the corneal apex.^8^ Therefore, the entrance pupil and plane of focus for the first Purkinje image are almost superimposed and can be focused at the same time. The distance between the camera and the eye was fixed, but additional control was achieved by tracking the size of the Purkinje image. In case of defocus (introduced by changing the distance), the Purkinje image becomes immediately larger. As the software determined the running standard deviation and average of the number of pixels in the Purkinje image and due to the very small depth of field of the camera, the distance between the camera and the eye was controlled to less than a millimetre. To determine the axial position of the COR behind the corneal apex, the distance from the corneal apex to the entrance pupil must be added.

### Experimental procedures

Experiments were conducted without spectacle correction. The screen used to display the targets was positioned so that the centre of the screen was in line with the centre of the pupil of the right eye for a straight‐ahead gaze position. The centre of rotation of the right eye was measured for two alternatingly presented horizontal fixation points (lateral positions right +11.9° and left –11.9°) and two alternatingly presented vertical fixation points (up +11.9°, down –11.9°). The left eye was covered using an eye patch. Head movements were restricted by a chin and head rest. Horizontal and vertical eye position changes were measured five times and angular data were averaged.

The study followed the tenets of the Declaration of Helsinki and was approved by the ethics commission of the Medical Faculty at the University of Tuebingen (Reference 400/2020BO). The nature of the experimental procedures was explained, and written informed consent was obtained from the subjects before taking the measurements.

## RESULTS

### Correlations between ocular biometry and refractive errors

**FIGURE 2:**
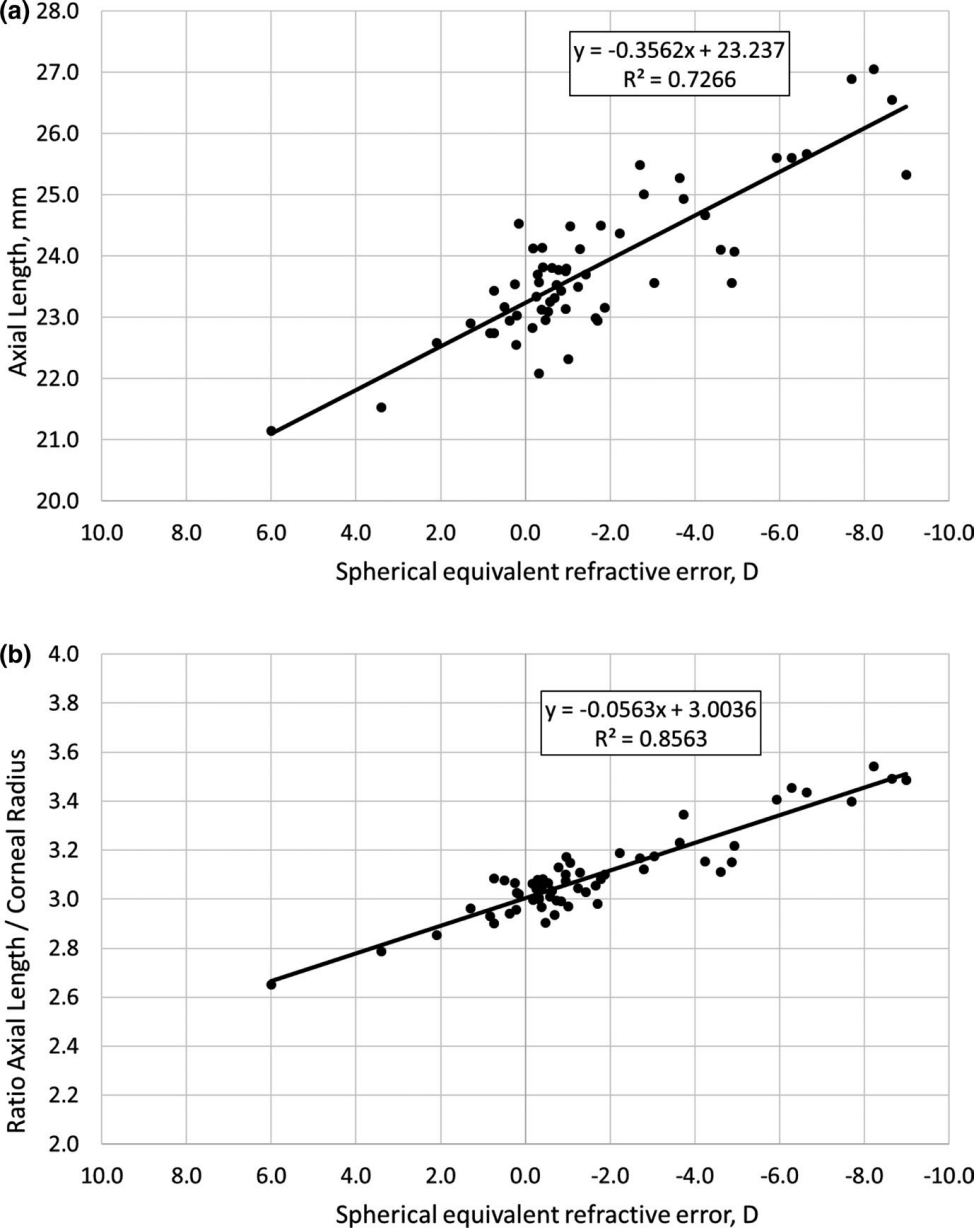
(a) Correlation between spherical equivalent refractive errors and axial lengths of the right eyes. (b) Spherical equivalent refractive errors plotted against AxL/CR. Note that the x‐axis is plotted in reverse order with hyperopia on the left and myopia on the right

In the 59 right eyes of 59 subjects, spherical equivalent refractive errors were significantly correlated with axial length (Figure 2a) with an *r* ^2^ value of 0.73. The slope of the regression showed that an increase in axial length of 1 mm caused 2.85 D more myopia. The correlation was further enhanced when the corneal radius of curvature was also considered. When the SE was plotted against the ratio of axial length and corneal radius (i.e., AxL/CR), the r^2^ value increased to 0.86, indicating that 86% of the refractive error could be explained by these two biometrical variables (Figure 2b).

### Axial position of the horizontal COR

**FIGURE 3:**
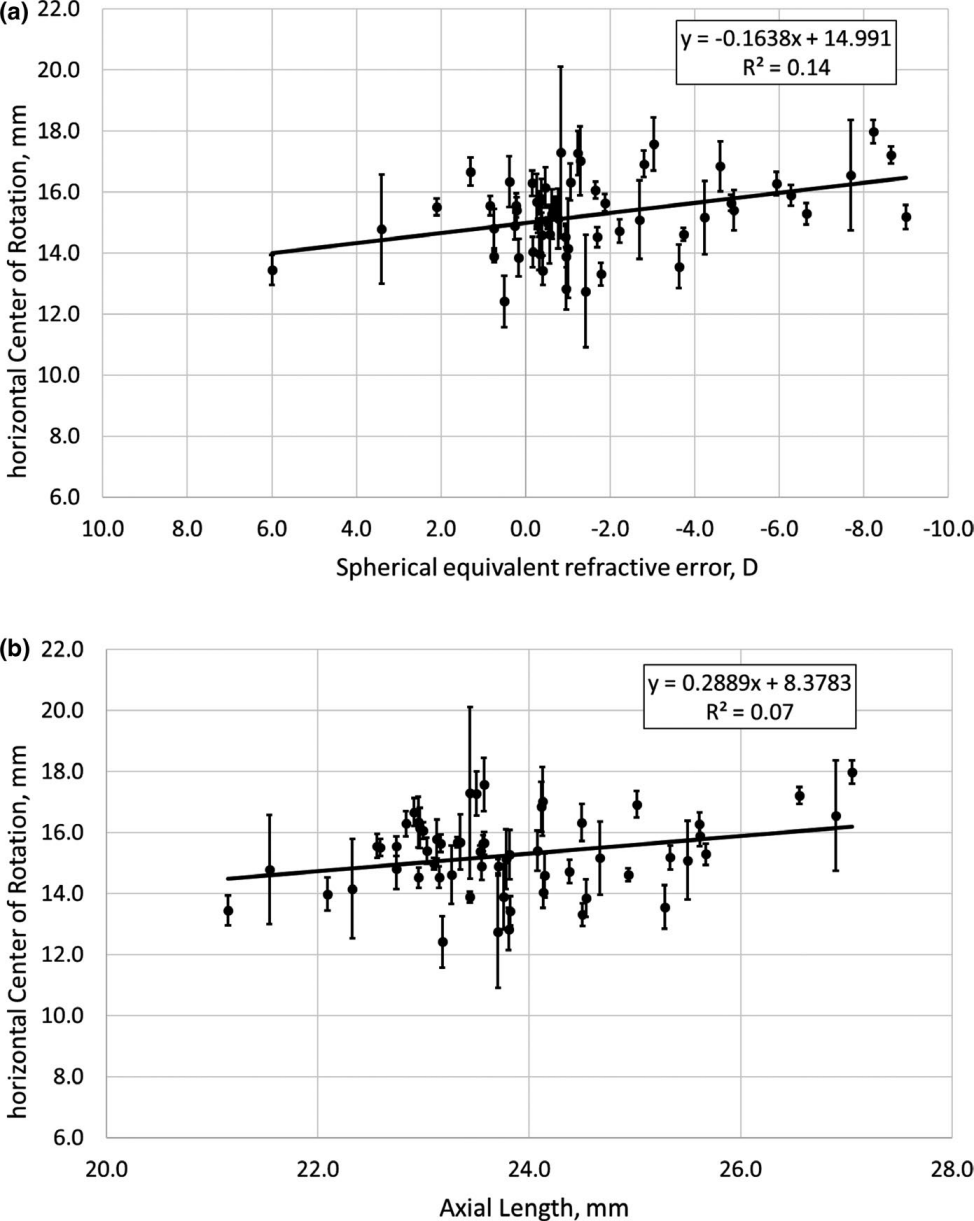
(a) Horizontal centre of rotation (COR) plotted as a function of spherical equivalent refractive error and (b) axial length. Note that in Figure 3a, the x‐axis is plotted in reverse order with hyperopia on the left and myopia on the right

On average, the COR in 59 subjects was located 15.3 ± 1.5 mm behind the corneal apex, with considerable variability ranging from 12.4 to 18.0 mm. Individual standard deviations of the five repeated measurements varied between ±0.17 and ±2.81 mm (see Figure 3). Analysis of Variance (ANOVA) and regression analysis revealed significant correlations between the locations of the COR and SE (horizontal COR vs. SE: *F*_1,57_ = 9.14, *p* = 0.004; *r* = 0.39, *p* = 0.90) and axial lengths (horizontal COR vs. axial length: *F*_1,57_ = 4.62, *p* = 0.03; *r* = 0.28, *p* = 0.02). The averaged individual horizontal COR, including the standard deviations, were plotted against SE (Figure 3a)) and axial length (Figure 3b).

### Axial position of the vertical COR

**FIGURE 4:**
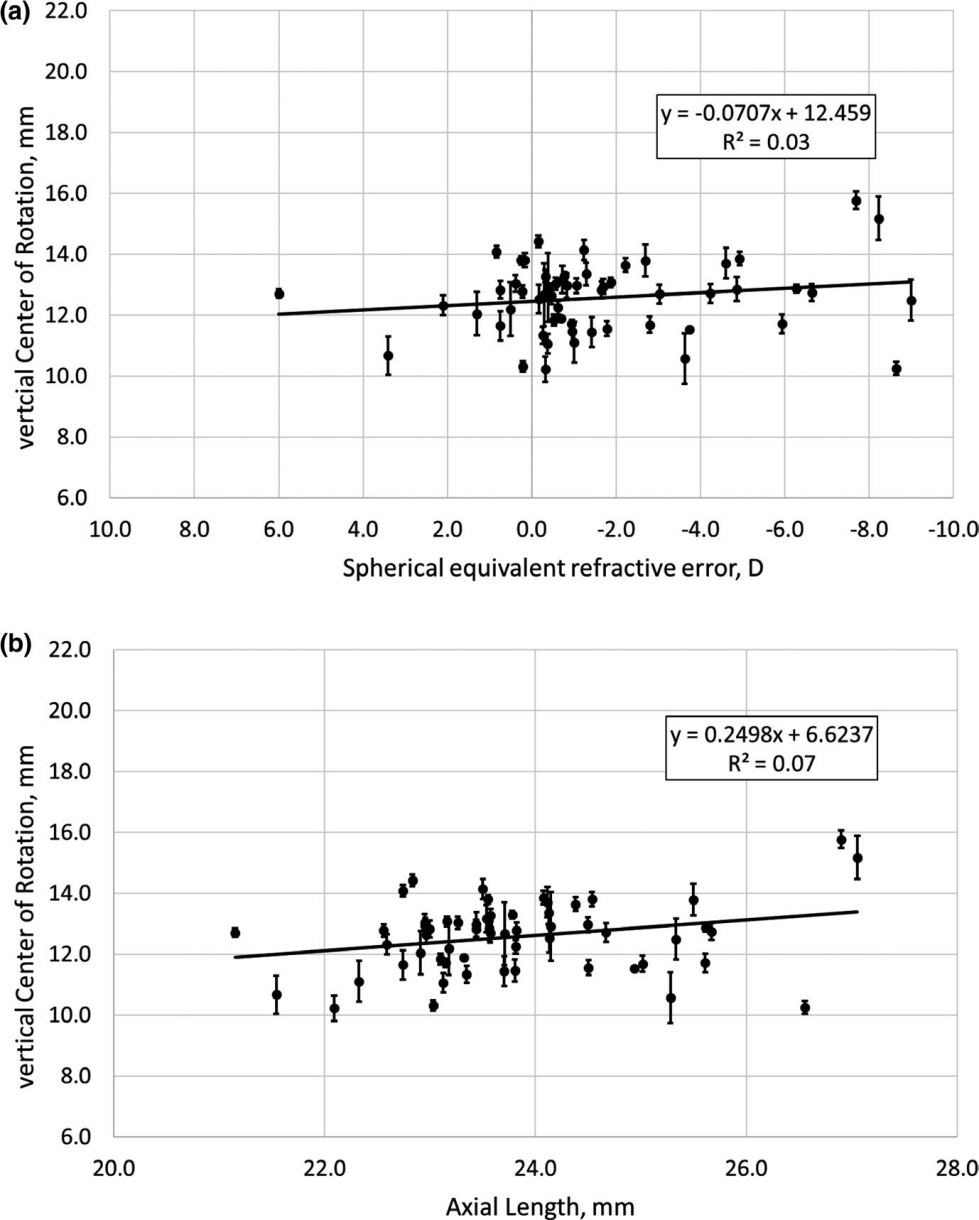
Vertical COR as a function of (a) spherical equivalent refractive error and (b) axial length. Note that in Figure 4a, the x‐axis is plotted in reverse order with hyperopia on the left and myopia on the right

The average axial position of the vertical COR was 12.5 ± 1.3 mm behind the corneal apex, ranging from 7.3 to 15.7 mm. Standard deviations of five repetitions of the measurement ranged from 0.1 to 1.1 mm. ANOVA and regression analysis revealed the vertical COR was not significantly related to refractive error (ANOVA: *F*_1,56_ = 1.77, *p* = 0.18; regression: *r* = 0.17, *p* = 0.9), but was associated with axial length (ANOVA: *F*_1,56_ = 4.03, *p* = 0.05; *r* = 0.25, *p* = 0.03). Results are shown in Figure 4a and b for vertical COR as a function of SE and axial length, respectively.

### Differences between horizontal and vertical COR

The CORs for horizontal and vertical eye movements differed by an average of 2.8 ± 1.5 mm, with the vertical COR located in front of the horizontal COR. This separation did not vary with SE (ANOVA: *F*_1,55_ = 2.33, *p* = 0.13; *r* = 0.2, *p* = 0.9) or axial length (ANOVA: *F*_1,55_ = 0.06, *p* > 0.99; *r* = 0.04, *p* = 0.4).

## DISCUSSION

### Relation of refractive error to ocular biometry

The correlation between refractive error and axial length observed in the current study matches previously published results.^9^ Also the enhanced correlation when the ratio of axial length to corneal radius of curvature was used rather than axial length alone was previously described for adult subjects, with *r*‐values of 0.6^10^ and 0.5^11^ being recorded. Recently, similar data were also observed in children (*r* = 0.81).^12^

### COR, axial length and refractive error

Earlier publications reported an average position for the horizontal COR in the range of 13.5 mm (emmetropes)^13^ to 14.5 mm (myopes)^13^ or even 15 mm^14^ behind the apex of the cornea. As refractive errors are primarily determined by axial length,^15^ it is reasonable to assume that the COR position may change with refractive error. It was also reported that the COR varies linearly with refractive error by 0.166 mm/D,^3^ or alternatively 0.14 and 0.18 mm for the horizontal and vertical COR, respectively.^2^ The current study does not reveal such a clear‐cut association. Significant interactions for the COR were only found for axial length but not for SE. A potential limitation in the current cohort was that axial lengths ranged from 21 to 27 mm but the majority of the SEs were between +1.0 D and −2.0 D. It is possible that the sample of subjects that led to a strong correlation between refractive error and the COR position in the earlier study^3^ had a more evenly distributed range of refractive errors.

### Potential link between COR, peripheral eye shape and peripheral refractive errors

While myopes typically show a more prolate eye shape and hyperopes an oblate shape,^16^ the relative peripheral refractive errors are more hyperopic in myopes and more myopic in hyperopes.^17^, ^18^ Furthermore, the vertical refraction profile was found to be flatter than the horizontal,^19^ although a recently published work showed smaller differences.^20^ Studying peripheral refractive errors with a scanning wavefront sensor over a 60 × 36° visual field in emmetropic children, Lan and colleagues found differences in refraction profiles in the horizontal and vertical meridians. While the horizontal meridian had a flat refraction profile in the majority of the children, the vertical meridian exhibited a myopic shift in the superior retina.^21^ Additionally, Pope et al. found differences in horizontal and vertical refractive errors between myopes and emmetropes that could be traced back to differences in retinal shape.^20^ However, at present, it remains speculative whether the observed differences in horizontal and vertical CORs result from different radii of curvature of the globe in the horizontal and vertical meridians.

### Horizontal and vertical COR

Fry and colleagues have already described a difference in the position of the COR for horizontal and vertical eye movements in a small group of six subjects, with an average COR of 14.9 and 12.3 mm for horizontal and vertical eye movements, respectively.^6^ Grolman measured the “sighting centre” in 50 subjects and found a difference of 1.4 mm between the horizontal (14.5 ± 0.55 mm) and vertical COR (13.1 ± 0.7 mm).^2^ In the current study, a difference between the horizontal and vertical COR was also found; however, it must be kept in mind that the angular amplitudes of the saccades were only 23.8° (2 × 11.9°). Potential lateral displacements of the COR have been ignored in currently available investigations. Additionally, oblique eye movements have not yet been studied, and it is likely that such examinations will shed more light on these questions.

### COR and ophthalmic lens design

The position of the COR of the eye with respect to the ophthalmic lens design will influence the ray bundles for different directions of gaze, as it acts as the “stop” position for such a lens. Simplifying these circumstances, Perches calculated sphero‐cylindrical errors for oblique viewing directions, different spherical corrections, different base curves and different positions of the COR of the eye, relative to the back vertex.^7^ The authors summarised that for an oblique viewing angle of 40°, a significant reduction in visual acuity was present in the case of back vertex powers of −2.0 D. Simulations with single vision lenses with spherical surfaces and powers of −1.5, −2.0 and −4.0 D for the participants in the present study revealed that areas of just noticeable or troublesome blur^22^ (compared to the habitual central correction) become elliptical instead of circular. These findings have implications for the optimisation of a single vision lens. Even for a purely spherical correction, the lens will no longer be rotationally symmetrical. Based on the findings regarding the different CORs for peripheral viewing in the horizontal and vertical directions, the optimal correction may not be achieved by a lens with an aspherical surface but rather one with an atoric surface. Whether these findings also have implications for lenses that are currently used to reduce myopia progression needs to be investigated. Current simulations do not consider the pantoscopic angle and degree of frame wrap that also influence the sphero‐cylindrical errors for the lens wearer.

### Possible limitations

The initial aim of the experiments was to develop a device to measure the COR reliably for horizontal and vertical viewing angles, and to investigate if there is a difference between these measurements that varies with the length of the eye. There have been multiple previous attempts to measure the position of the COR. For example, Fry and Hill^3^ asked their subjects to fixate a target at different visual angles, ranging from +40 to −40° in the horizontal plane. Connecting the fixation points with the centre of the pupil at the different fixation positions, they determined the intersection of these lines as the COR. Park and Park^4^ used a similar procedure, but head movements and fixation were controlled better. Both groups differed in their interpretation as to whether the COR contains stationary (Fry and Hill) or translatory components (Park and Park). In the current study, we found no consistent translational component for the COR, but only horizontal and vertical directions were studied and it may be that oblique angles of fixation may cause more complicated shifts in the position of the COR. Our set‐up also differed from these previous investigations as a camera was used to track the pupil centre in real‐time, and to calculate COR position relative to the corneal apex from the lateral displacements of the pupil centre using simple trigonometry. A complication arose from the fact that the camera was not positioned in the horizontal plane, but was 23.8° below this plane to ensure visibility of the targets. However, our software corrected for the parallax resulting from oblique imaging of the pupil centre position when the eye was turned to the periphery. Another problem might be the increasingly elliptical shape of the pupil for peripheral targets. However, for two reasons, this was not critical: (1) the pupil centre was still correctly detected by our imaging processing algorithm as it did not involve circle fitting and (2) the peripheral fixation angles were small (+11°), causing only minimal distortions of pupil shape. The viewing angles for peripheral targets were, however, large enough to obtain reliable estimates of the COR, as can be seen from the standard deviations of the repeated measurements. For reliable estimates of eye position during fixation, note that our software accepted fixation only when the running standard deviation dropped below 0.5°. As described above, the final axial position of the COR behind the corneal apex was calculated by adding the distance from the corneal apex to the entrance pupil, and a constant value of 3.6 mm was chosen. It is known that corneal radii of curvature vary among subjects. For instance, in a subject with a corneal radius of 8 mm, the plane of focus of the first Purkinje image would be 4 mm behind the corneal apex instead of the value of 3.6 mm used in the current setup. However, the plane of the entrance pupil would also be displaced more distally due to the longer focal length of the cornea. Therefore, the first Purkinje image would still remain close to the plane of the entrance pupil, although the distance to the corneal apex would be 0.2 mm larger than in the current setup. However, one should keep in mind that the difference of 0.2 mm (per our manuscript) would only be about 1.5% for an average horizontal COR of 15 mm and 2% for an average vertical COR of 13 mm, which is small compared to the variability that we found among subjects (about 10%).

## CONFLICTS OF INTEREST

AO and SW are paid employees of Carl Zeiss Vision International GmbH / MB. FS receives financial support from Carl Zeiss Vision International GmbH. There are no patents, products in development or marketed products associated with this research to declare. The sponsor had no specific role in the production of the paper (e.g. writing, analysis or control over publication). This research received no specific grant from any funding agency in the public, commercial, or not‐for‐profit sectors.

## AUTHOR CONTRIBUTION

**Arne Ohlendorf:** Conceptualization (lead); Data curation (lead); Formal analysis (lead); Investigation (lead); Writing – original draft (lead); Writing – review & editing (equal). **Frank Schaeffel:** Methodology (equal); Software (lead); Validation (equal); Writing – review & editing (equal). **Siegfried Wahl:** Project administration (lead); Resources (lead); Writing – review & editing (equal).

## References

[1] Squire LR , Bloom FE , Spitzer NC , et al. Versional movements shift the line of gaze. Fundam Neurosci 2008;1:135–51.

[2] Grolman B . The sighting center. Am J Optom Arch Am Acad Optom 1963;40:666–75. 14077197 10.1097/00006324-196311000-00002

[3] Fry G , Hill W . Center of rotation of the eye. Am J Optom Arch Am Acad Optom 1962;39:581–95. 13959969 10.1097/00006324-196211000-00001

[4] Park RS , Park GE . The center of ocular rotation in the horizontal plane. Am J Physiol 1933;104:545–52.

[5] Perkins ES , Hammond B , Milliken AB . Simple method of determining the axial length of the eye. Br J Ophthalmol 1976;60:266–70. 1276113 10.1136/bjo.60.4.266 PMC1017489

[6] Fry GA , Hill WW . The mechanisms of elevating the eye. Am J Optom Arch Am Acad Optom 1963;40:707–16. 14088686 10.1097/00006324-196312000-00001

[7] Perches S , Ares J , Collados V , Palos F . Sphero‐cylindrical error for oblique gaze as a function of the position of the centre of rotation of the eye. Ophthalmic Physiol Opt 2013;33:456–66. 23786385 10.1111/opo.12077

[8] Howland HC , Sayles N . Photokeratometric and photorefractive measurements of astigmatism in infants and young children. Vision Res 1985;25:73–81. 3984220 10.1016/0042-6989(85)90082-3

[9] Kim HS , Yu DS , Cho HG , Moon BY , Kim SY . Comparison of predicted and measured axial length for ophthalmic lens design. PLoS One 2019;14:1–12. 10.1371/journal.pone.0210387 PMC6322735 30615674

[10] Hashemi H , Khabazkhoob M , Miraftab M , et al. Axial length to corneal radius of curvature ratio and refractive errors. J Ophthalmic Vis Res 2013;8:220–6. 24349665 PMC3853782

[11] Badmus SA , Ajaiyeoba AI , Adegbehingbe BO , Onakpoya OH , Adeoye AO . Axial length/corneal radius of curvature ratio and refractive status in an adult Nigerian population. Niger J Clin Pract 2017;20:1328–34. 29192640 10.4103/njcp.njcp_183_16

[12] He X , Zou H , Lu L , et al. Axial length/corneal radius ratio: Association with refractive state and role on myopia detection combined with visual acuity in Chinese schoolchildren. PLoS One 2015;10:1–19. 10.1371/journal.pone.0111766 PMC4333577 25693186

[13] Von Noorden GK , Campos E . Physiology of the ocular movements. Binocul Vis Ocul Motil 2002;36:44–5.

[14] Alda J , Alonso J . Ophthalmic optics. Encycl Opt Eng 2011;1:1563–76.

[15] Meng W , Butterworth J , Malecaze F , Calvas P . Axial length of myopia: a review of current research. Ophthalmologica 2011;225:127–34. 20948239 10.1159/000317072

[16] Atchison DA , Jones CE , Schmid KL , et al. Eye shape in emmetropia and myopia. Investig Ophthalmol Vis Sci 2004;45:3380–6. 15452039 10.1167/iovs.04-0292

[17] Seidemann A , Schaeffel F , Guirao A , Lopez‐Gil N , Artal P . Peripheral refractive errors in myopic, emmetropic, and hyperopic young subjects. J Opt Soc Am A 2002;19:2363–73. 10.1364/josaa.19.002363 12469730

[18] Wallman J , Winawer J . Homeostasis of eye growth and the question of myopia. Neuron 2004;43:447–68. 15312645 10.1016/j.neuron.2004.08.008

[19] Atchison DA , Jones CE , Schmid KL , et al. Eye shape in emmetropia and myopia. Invest Ophthalmol Vis Sci 2004;45:3380–6. 15452039 10.1167/iovs.04-0292

[20] Pope JM , Verkicharla PK , Sepehrband F , et al. Three‐dimensional MRI study of the relationship between eye dimensions, retinal shape and myopia. Biomed Opt Express 2017;8:2386. 10.1364/BOE.8.002386 PMC5480487 28663880

[21] Lan W , Lin Z , Yang Z , Artal P . Two‐dimensional peripheral refraction and retinal image quality in emmetropic children. Sci Rep 2019;9:1–9. 10.1038/s41598-019-52533-7 31700132 PMC6838170

[22] Atchison DA , Fisher SW , Pedersen CA , Ridall PG . Noticeable, troublesome and objectionable limits of blur. Vision Res 2005;45:1967–74. 15820515 10.1016/j.visres.2005.01.022

